# Modulation of Simulated Microgravity-Induced Behavioral Responses by Caffeine in Zebrafish: A Pilot Study

**DOI:** 10.3390/mps9050145

**Published:** 2026-10-08

**Authors:** Cǎtǎlina Ionescu, Irene Di Domenicantonio, Franco Cardone, Marco Sbriccoli, Alin Constantin Pînzariu, Bogdan Novac, Otilia Novac, Mihai Hogas, Alin Ciobîcă

**Affiliations:** 1 Doctoral School of Biology, Faculty of Biology, Alexandru Ioan Cuza University of Iasi, Bd. Carol I no. 20A, 700505 Iasi, Romania; catalinaionescu81@yahoo.com; 2 “Ioan Haulica” Institute, Apollonia University, Pacurari Street 11, 700511 Iasi, Romania; alin.ciobica@uaic.ro; 3 Core Facilities Technical-Scientific Service, Istituto Superiore di Sanità, 00161 Rome, Italy; irenedido.96@gmail.com (I.D.D.); franco.cardone@iss.it (F.C.); 4 Department of Neuroscience, Istituto Superiore di Sanità, 00161 Rome, Italy; marco.sbriccoli@iss.it; 5 Faculty of Medicine, Grigore T. Popa University of Medicine and Pharmacy, 700115 Iasi, Romania; otilia.novac@umfiasi.ro (O.N.); mihai.hogas@umfiasi.ro (M.H.); 6 Department of Biology, Faculty of Biology, Alexandru Ioan Cuza University of Iasi, Bd. Carol I no. 20A, 700505 Iasi, Romania; 7 “Olga Necrasov” Center, Department of Biomedical Research, Romanian Academy, 700481 Iasi, Romania; 8 CENEMED Platform for Interdisciplinary Research, Grigore T. Popa University of Medicine and Pharmacy, 700115 Iasi, Romania

**Keywords:** zebrafish, simulated microgravity, caffeine, clinostat, locomotor performance, novel tank test

## Abstract

This pilot study evaluates the effects of acute caffeine administration on adult zebrafish behavioral responses under simulated microgravity, aiming to understand how common dietary stimulants modulate neurobehavioral adaptations in altered-gravity environments. Adult zebrafish were immersed in 25 mg/L caffeine for 30 min, followed by a 1 h exposure to simulated microgravity using a clinostat system, before immediate assessment in a standard locomotor test and a vertical Novel Tank Test tracked via EthoVision XT 14 software. While independent exposures to caffeine or microgravity yielded minimal modifications to baseline swimming kinetics, their simultaneous application resulted in an increased hyperlocomotor response, characterized by increased distance moved, average velocity, and moving duration. Co-exposure also altered stereotypic motor coordination, significantly enhancing clockwise rotations. Furthermore, dual-view heat maps in the Novel Tank Test showed that the simultaneous exposure to caffeine and microgravity showed altered wall-associated spatial behavior (thigmotaxis) and bottom-dwelling avoidance, shifting behavioral output toward active vertical exploration and central arena habituation. These results suggest that simulated microgravity may influence the psychopharmacological expression of caffeine, with the combined exposure being associated with altered vertical exploration and motor activity, thereby supporting the potential utility of adult zebrafish as an experimental model for space biomedical research and highlighting the complex role of dietary countermeasures in altered-gravity environments.

## 1. Introduction

Spaceflight exposes organisms to a unique combination of environmental stressors, among which microgravity represents one of the most disruptive factors affecting physiological and neurobehavioral homeostasis. Both real and simulated microgravity have been shown to induce alterations in cardiovascular regulation, musculoskeletal integrity, neurovestibular function, and behavior, largely through changes in sensory input integration and stress-response systems. These adaptations are particularly relevant for long-duration missions, where cumulative physiological dysregulation may compromise performance and health of astronauts [1,2,3].

At the neurobehavioral level, exposure to altered-gravity conditions has been associated with disruptions in locomotor activity, anxiety-related behavior, and sensorimotor coordination across multiple model organisms, highlighting the central role of gravity-dependent cues in maintaining neural circuit stability [4,5,6,7]. In this context, the identification of safe and effective countermeasures has become a major focus in space biomedical research. Nutritional strategies represent one of the most practical and continuously available interventions during space missions, as diet is already an essential component of astronaut health maintenance and is known to influence cognition, fatigue, and stress resilience [8].

Among dietary bioactive compounds, caffeine is one of the most widely consumed psychoactive substances on Earth and is also present in spaceflight nutrition systems. Its primary mechanism of action involves antagonism of adenosine receptors, leading to increased arousal, enhanced alertness, and modulation of central nervous system activity [9]. Beyond its stimulatory effects, caffeine has been investigated for its ability to influence stress responsiveness and behavioral performance under challenging conditions, although its role as a potential modulator of spaceflight-related stressors remains insufficiently understood [10].

Experimental evidence from aquatic vertebrate models indicates that caffeine can significantly alter locomotor and anxiety-like behaviors in a dose-dependent manner, suggesting its capacity to modulate neural circuits involved in stress and exploration [11,12]. In zebrafish, caffeine exposure has been associated with changes in locomotion, shoaling behavior, and anxiety-related endpoints, reinforcing its relevance as a neuroactive compound in behavioral research [13,14].

The zebrafish (*Danio rerio*) has emerged as a powerful vertebrate model for studying neurobehavioral and toxicological effects of pharmacological agents, including psychoactive substances. Its genetic tractability, conserved neurobiology with mammals, transparent embryonic development, and well-characterized behavioral repertoire make it particularly suitable for assessing stress-related phenotypes. Importantly, zebrafish have been extensively used in studies investigating both caffeine exposure and environmental stressors, supporting their potential translational relevance for neurobehavioral research [15,16].

Given the increasing interest in identifying simple and effective countermeasures against spaceflight-induced stressors, understanding how caffeine modulates behavior under simulated microgravity conditions is of both fundamental and applied importance. In this context, the present pilot study investigates the effects of caffeine on behavioral responses in zebrafish exposed to simulated microgravity, aiming to explore its potential as a modulatory agent in altered-gravity environments. Behavioral endpoints are particularly relevant in this framework, as they provide an integrative readout of neurophysiological function and stress adaptation, reflecting the overall functional state of the organism under environmental perturbation.

## 2. Materials and Methods

### 2.1. Ethical Note

Animals were treated and maintained in accordance with the EU Commission Recommendation (2007), Directive 2010/63/EU of the European Parliament and of the Council on the protection of animals used for scientific purposes. All experimental procedures complied with institutional and international guidelines for the care and use of laboratory animals. The protocol was approved by the Ethics Committee of the Faculty of Biology, “Alexandru Ioan Cuza” University, Iași, Romania (No. 692/11.02.2026).

### 2.2. Animal Maintenance

For the present study, adult wild-type AB zebrafish (*Danio rerio*), approximately 9–10 months old, were obtained from an authorized local breeder. Fish were acclimatized under standard laboratory conditions for 10 days prior to experimentation in 10 L aquaria equipped with continuous aeration and dechlorinated water, which was renewed daily. Environmental conditions were maintained as follows: temperature 26 ± 1 °C, pH 7.4–7.6, dissolved oxygen 7.0–7.5 mg/L, ammonia < 0.005 ppm, and conductivity approximately 500 µS. Housing density was maintained at approximately 1 fish per liter. Fish were fed twice daily with commercial flake food (Tetramin^®^, Tetra, Melle, Germany; ~2–3 mm diameter), and feeding was standardized across all groups and performed at consistent times relative to experimental procedures. The light/dark cycle was maintained at 12:12 h. Sex was identified visually and groups were balanced for male/female ratio. After acclimatization, fish were randomly assigned to experimental groups (*n* = 6).

### 2.3. Substance and Treatment

Caffeine (≥99% purity, Sigma-Aldrich, Merck KGaA, Darmstadt, Germany) was used for pharmacological exposure. A stock solution was freshly prepared in dechlorinated water and diluted to a final concentration of 25 mg/L, selected based on a previous zebrafish study reporting behavioral effects at this concentration [17].

Caffeine exposure was performed via immersion for 30 min. Following caffeine exposure, fish in the relevant groups were subjected to simulated microgravity using an improvised clinostat system based on established literature for gravitational unloading in aquatic models [18,19,20,21]. These references provide methodological precedent for the application of clinorotation as a ground-based approach to gravitational unloading in aquatic models and are not intended to imply that clinorotation itself induces a stress response.

The clinostat is an electromechanical device widely utilized in ground-based space biology to counteract the directional effects of Earth’s gravity (1g) through continuous rotation. Its operating principle relies on the constant, slow reorientation of the biological model around an axis, thereby randomizing the gravitational vector over time. This multi-directional redistribution prevents the organism’s gravity-sensing systems from perceiving a fixed gravitational pull, achieving a state of functional weightlessness or gravitational unloading. In aquatic models, this continuous rotation alters swimming dynamics and disrupts neurovestibular inputs, effectively simulating the sensory and physiological challenges of real microgravity. Adult zebrafish were placed in a circular experimental container with an internal diameter of approximately 16 cm and an internal depth of approximately 5 cm, containing approximately 1000 mL of dechlorinated water. The container was completely filled with water and sealed before exposure, with no residual air bubbles or air–water interface. Six adult zebrafish belonging to the same experimental group were placed simultaneously in the container for each exposure. The exposure was conducted under the same temperature conditions used for animal maintenance (26 ± 1 °C).

The sealed container was mounted on the clinostat and continuously rotated around a horizontal axis at 30 rpm for 1 h. Comparable short-term clinorotation protocols, including 60 min exposure, have previously been shown to induce measurable biological responses in aquatic organisms [22].

Because the model involved freely swimming adult zebrafish in a water-filled container, continuous rotation may also have generated water movement and associated mechanical stimulation. These potential rotation-associated hydrodynamic effects cannot be completely excluded; therefore, the exposure should not be considered fully equivalent to true microgravity.

Behavioral testing was performed immediately after exposure to assess the acute effects of caffeine and simulated-microgravity exposure.

### 2.4. Experimental Design

Zebrafish were exposed to caffeine (25 mg/L) [17] for 30 min via immersion, followed by exposure to simulated microgravity conditions for a 1 h interval.

Fish were randomly distributed into experimental groups, including a control group maintained under standard gravity conditions without caffeine exposure and an experimental group exposed to caffeine followed by simulated microgravity.

**Figure 1:**
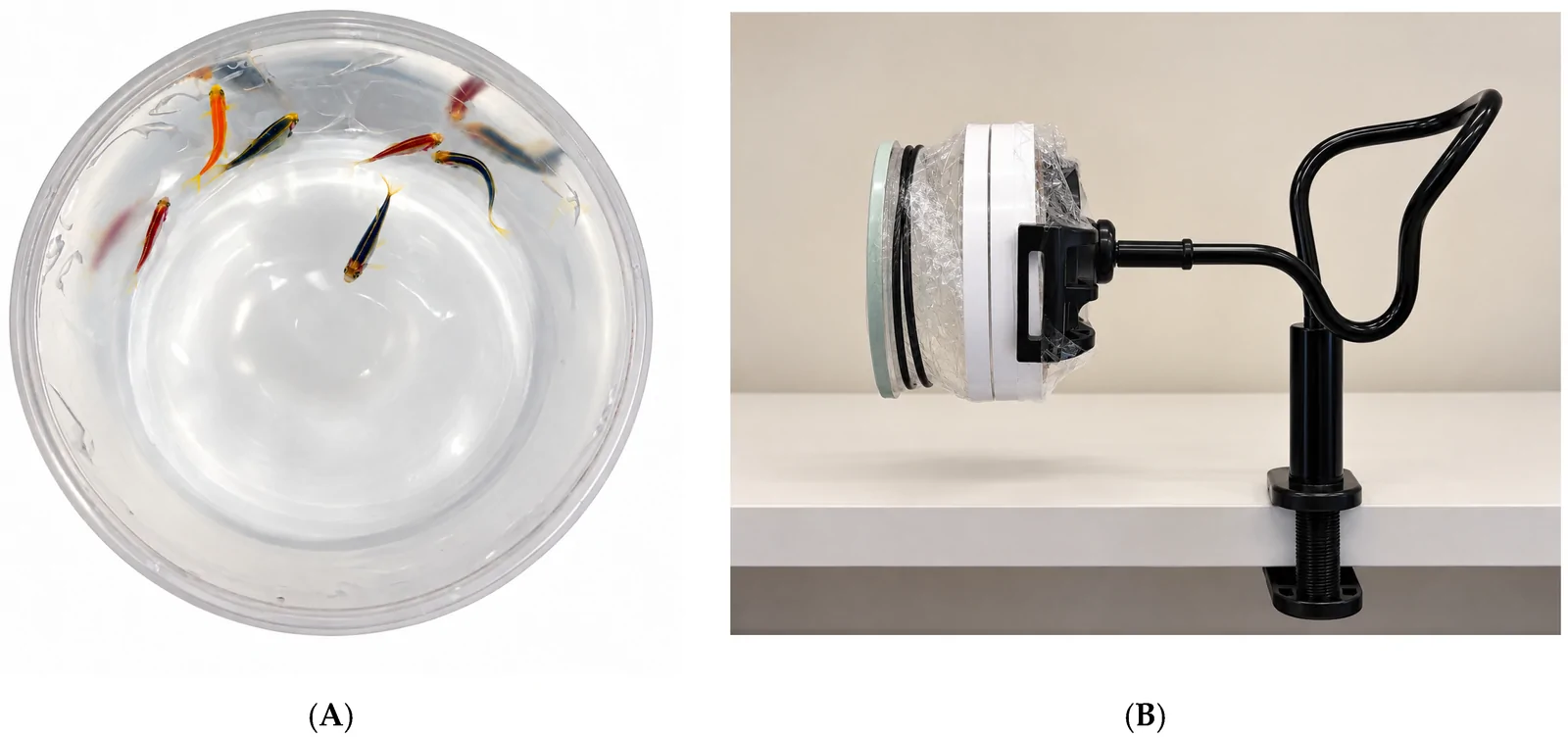
Experimental setup for simulated microgravity exposure. (**A**) Zebrafish specimens placed in a circular plastic container under control (static) conditions; (**B**) the experimental container mounted onto a clinostat for simulated microgravity conditions via continuous rotation (original photos).

During the corresponding 1 h exposure period, control fish remained in their standard maintenance aquarium under normal 1g conditions. They were not placed in the experimental container or mounted on the clinostat and were not exposed to motor operation or rotation. Therefore, this group represents a static 1g control rather than a rotation-matched control (Figure 1).

Behavioral responses were assessed immediately after exposure using locomotor activity analysis and the Novel Tank Test (NTT). To ensure high-throughput accuracy and eliminate observer bias, video acquisition and automated tracking were performed using the EthoVision XT video tracking system (Noldus Information Technology, Wageningen, The Netherlands). Individual fish from each group were gently transferred into identical, transparent novel test tanks filled with standard system water. The arena setup was illuminated uniformly to optimize contrast, and behavioral profiles were recorded via a high-resolution digital camera positioned directly in front of the tanks for a fixed observation period.

Using the EthoVision XT 14 software interface, each tank was digitally mapped and divided into distinct virtual zones, specifically a bottom zone (to evaluate thigmotaxis and anxiety-like diving responses) and a top zone (to monitor exploratory tendencies). The software tracked the center point of each zebrafish in real time, extracting key locomotor and behavioral endpoints. General motor function was quantified through total distance traveled (cm) and mean velocity (cm/s), while anxiety-like behavior was evaluated based on the latency to enter the top zone, total time spent in the top vs. bottom sections, and the cumulative duration of freezing bouts.

### 2.5. Behavioural Tests

#### 2.5.1. Locomotor Activity Assessment

The locomotor activity assessment was employed to evaluate the potential disruptions in motor performance induced by microgravitational stress, as well as to investigate whether caffeine administration could modulate these effects. In this study, each zebrafish was individually placed in a standard observation tank positioned on a uniform white surface, with external walls shielded to eliminate confounding visual stimuli and optimize contrast for digital tracking.

Swimming performance and basal exploratory behavior were recorded for a continuous duration of 5 min per fish using a high-resolution top-view camera system. The recorded video trajectories were automatically quantified and analyzed using EthoVision XT version 14 software (Noldus Information Technology, Wageningen, The Netherlands). To obtain a comprehensive profile of locomotor performance and physiological vitality, the following parameters were systematically evaluated: total distance moved (cm), average swimming velocity (cm/s), total mobility duration (s), as well as the frequency and cumulative duration of immobility episodes.

#### 2.5.2. The Novel Tank Test

The novel tank test considers new environments to be anxiogenic for zebrafish, inducing initial occupation of the tank bottom followed by progressive habituation and increased exploration of the upper zone [23,24]. In this study, each zebrafish was individually transferred to a simple tank placed on a white bench, with walls and back covered with dull white paper to facilitate tracking. The tank was filled with a water column suitable for swimming, and no internal or external enrichment was present.

Swimming behaviour and space occupation were recorded for 5 min using a top-view and a side-view camera, and trajectories were analyzed using EthoVision XT version 14 (Noldus Information Technology, Wageningen, The Netherlands). The tank was virtually divided into upper (UP) and bottom (BTM) zones, and the following parameters were measured: distance moved (cm); velocity (cm/s); inactivity time (s); transitions to the bottom half (latency); time spent in the bottom half (s); time spent near the walls (s); clockwise rotations (frequency); and counter-clockwise rotations (frequency).

### 2.6. Statistical Analysis

Data were analyzed using GraphPad Prism 10.4.1 (GraphPad Software, San Diego, CA, USA). Normality was assessed using the Shapiro–Wilk test. Statistical differences between groups were evaluated using two-way ANOVA followed by Šídák’s multiple-comparisons test. Results are expressed as mean ± standard deviation (SD), and statistical significance was set at *p* < 0.05.

## 3. Results

### 3.1. Locomotor Performance Test

During the locomotor performance test, behavioral parameters were analyzed to evaluate the impact of microgravitational stress on the swimming performance of zebrafish, as well as to determine whether caffeine administration modulates these responses.

**Figure 2:**
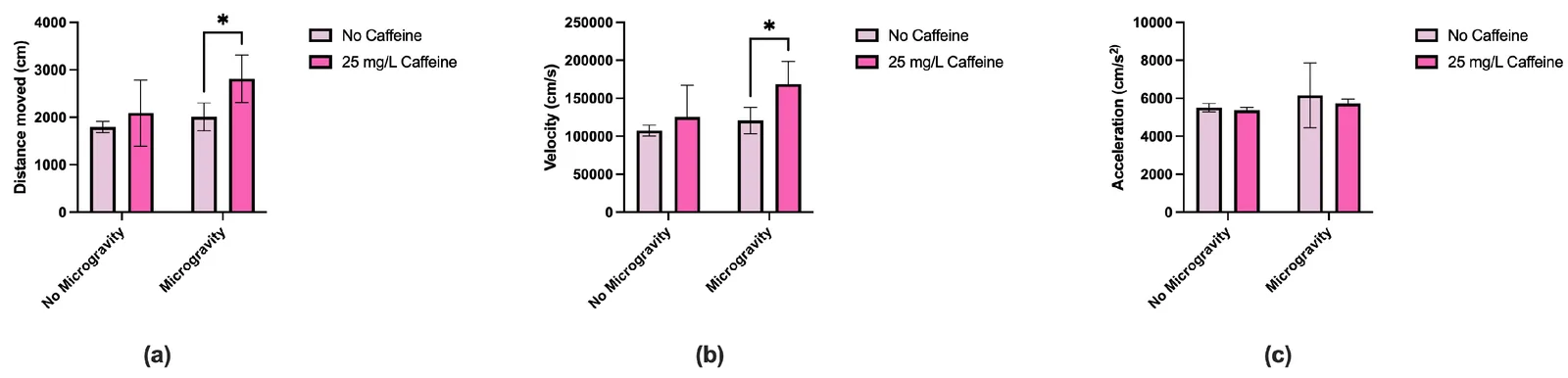
Acute effects of caffeine (25 mg/L) alone and in combination with simulated microgravity on adult zebrafish locomotor performance. Behavioral parameters measured include: (**a**) distance moved, (**b**) velocity, and (**c**) acceleration. Data are presented as mean ± SD and were analyzed using two-way ANOVA followed by Šídák’s multiple-comparisons test. Statistically significant differences are indicated as follows: * *p* < 0.05.

The total distance moved was analyzed as a primary parameter of locomotor activity and exploratory behavior (Figure 2a). Two-way ANOVA revealed significant main effects of microgravity (*p* = 0.04) and caffeine (*p* = 0.02), whereas the caffeine + microgravity interaction was not significant (*p* = 0.24). Šídák’s multiple-comparisons test showed that caffeine significantly increased the distance moved under microgravity conditions (*p* = 0.03), whereas no significant caffeine-induced change was observed under no-microgravity conditions (*p* = 0.53). These findings indicate increased locomotor activity following combined caffeine and microgravity exposure; however, the absence of a significant interaction does not support a synergistic effect between the two factors.

The average velocity was further examined as an indicator of swimming intensity and locomotor performance (Figure 2b). Two-way ANOVA similarly revealed significant main effects of microgravity (*p* = 0.04) and caffeine (*p* = 0.02), while the caffeine + microgravity interaction was not significant (*p* = 0.24). Šídák’s multiple-comparisons test showed no significant effect of caffeine under no-microgravity conditions (*p* = 0.53), whereas caffeine significantly increased swimming velocity under microgravity conditions (*p* = 0.03). Thus, the combined caffeine and microgravity condition was associated with increased swimming velocity, although the absence of a significant interaction does not support interpretation of this effect as synergistic.

Acceleration was evaluated as an indicator of rapid changes in swimming movement and locomotor reactivity (Figure 2c). Two-way ANOVA revealed no significant caffeine + microgravity interaction (*p* = 0.71) and no significant main effects of either microgravity (*p* = 0.22) or caffeine (*p* = 0.47). Consistently, Šídák’s multiple-comparisons test showed no significant effect of caffeine under either no-microgravity (*p* = 0.96) or microgravity conditions (*p* = 0.69). These findings indicate that acceleration was not significantly affected by caffeine, simulated microgravity, or their combined exposure under the experimental conditions tested.

**Figure 3:**
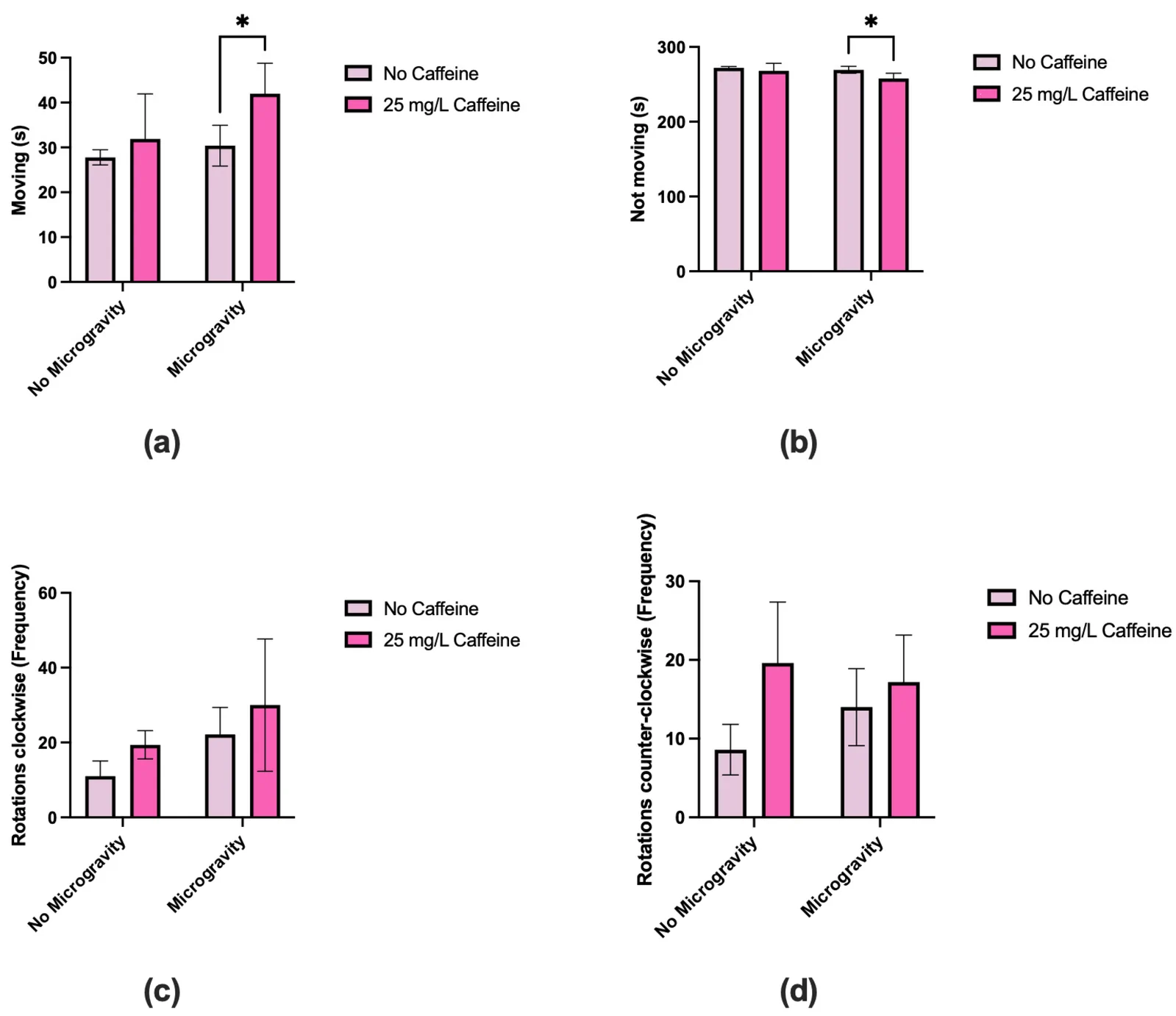
Acute effects of caffeine (25 mg/L) alone and in combination with microgravity on zebrafish kinetic state durations and turning frequencies during the locomotor performance test. Behavioral parameters measured include: (**a**) moving duration, (**b**) not moving duration, (**c**) clockwise rotations and (**d**) counter-clockwise rotations. Data are presented as mean ± SD and were analyzed using two-way ANOVA followed by Šídák’s multiple-comparisons test. Statistically significant differences are indicated as follows: * *p* < 0.05.

The moving duration was quantified as an indicator of the time spent in an active swimming state. Two-way ANOVA revealed significant main effects of microgravity (*p* = 0.05) and caffeine (*p* = 0.02), whereas the caffeine + microgravity interaction was not significant (*p* = 0.22). Šídák’s multiple-comparisons test showed no significant effect of caffeine under no-microgravity conditions (*p* = 0.55), while caffeine significantly increased moving duration under microgravity conditions (*p* = 0.02). These findings indicate increased active locomotion following caffeine exposure under microgravity conditions. However, because the caffeine + microgravity interaction was not significant, the results do not provide statistical evidence for a synergistic interaction between caffeine and microgravity (Figure 3a).

The not-moving duration was evaluated as an indicator of locomotor inactivity. Two-way ANOVA revealed significant main effects of microgravity (*p* = 0.04) and caffeine (*p* = 0.02), whereas the caffeine + microgravity interaction was not significant (*p* = 0.23). Šídák’s multiple-comparisons test showed no significant effect of caffeine under no-microgravity conditions (*p* = 0.56), whereas caffeine significantly reduced not-moving duration under microgravity conditions (*p* = 0.03). These findings indicate reduced locomotor inactivity following caffeine exposure under microgravity conditions, consistent with an overall increase in locomotor activity. Nevertheless, the absence of a significant interaction does not support a synergistic effect between caffeine and microgravity (Figure 3b).

The frequency of clockwise rotations was evaluated to characterize directional rotational behavior. Two-way ANOVA revealed a significant main effect of microgravity (*p* = 0.03), whereas the main effect of caffeine (*p* = 0.09) and the caffeine + microgravity interaction (*p* = 0.95) were not significant. Šídák’s multiple-comparisons test detected no significant effect of caffeine under either no-microgravity (*p* = 0.36) or microgravity conditions (*p* = 0.41). Thus, microgravity significantly influenced clockwise rotational behavior overall, with no evidence that caffeine modified this effect (Figure 3c).

For counter-clockwise rotations, two-way ANOVA revealed a significant main effect of caffeine (*p* = 0.01), whereas the main effect of microgravity (*p* = 0.56) and the caffeine + microgravity interaction (*p* = 0.15) were not significant. Šídák’s multiple-comparisons test showed that caffeine significantly increased counter-clockwise rotations under no-microgravity conditions (*p* = 0.02), but not under microgravity conditions (*p* = 0.63). Thus, caffeine significantly influenced counter-clockwise rotational behavior overall, without evidence of a caffeine + microgravity interaction (Figure 3d).

**Figure 4:**
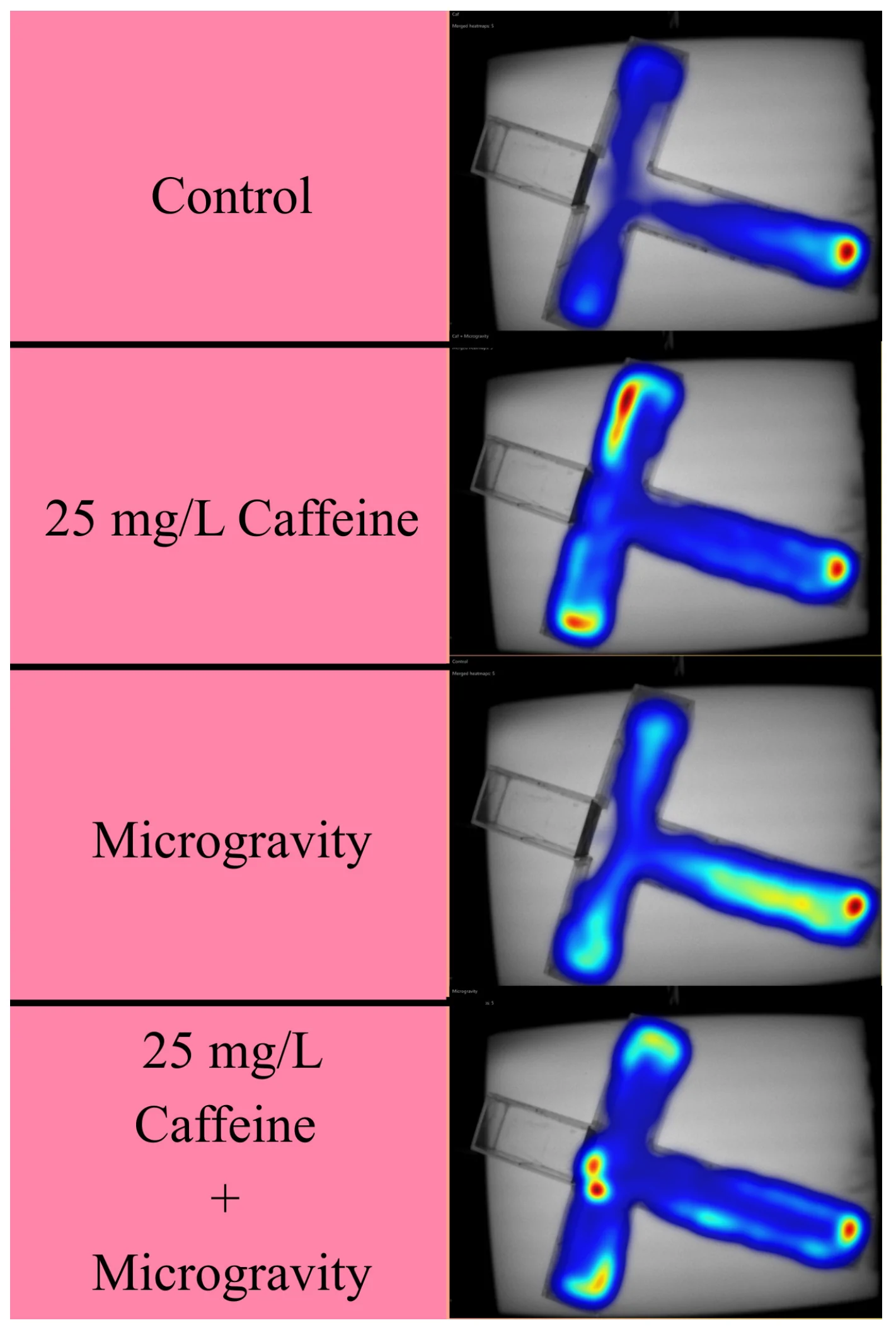
Representative EthoVision XT heat maps illustrating the spatial occupancy and locomotor distribution of adult zebrafish during the locomotor performance assessment in response to 25 mg/L caffeine, microgravity, and their combination. The color spectrum ranges from blue (low occupancy/transient transition) to red/yellow (high occupancy/extended duration).

To provide a qualitative visualization of spatial occupancy patterns, representative behavioral heat maps were generated across the experimental groups (Figure 4). The baseline control cohort exhibited a highly localized residency pattern, characterized by a single prominent high-density hotspot (red/yellow signal) restricted to the distal extremity of one arm, reflecting a conservative exploratory profile or prolonged occupancy in a single zone. Conversely, acute administration of 25 mg/L caffeine was associated with a broader distribution of high-occupancy zones, as evidenced by intense, widespread heat signals at multiple arm extremities, suggesting a more spatially distributed exploratory pattern in the representative trajectory. Exposure to simulated microgravity induced a distinct architectural shift in tracking density, presenting a continuous, elongated yellow-green trajectory along the main corridor of the arena. This phenotypic pattern points toward persistent shuttling or stereotypic loop-like swimming behavior along the long axis, consistent with an altered locomotor pattern under simulated microgravity. Notably, the combined 25 mg/L caffeine + microgravity treatment group displayed a profound redistribution of spatial exploration. This co-exposure group was characterized by the emergence of multiple intense focal hotspots, including pronounced density clusters situated precisely at the critical central intersection and bifurcation points of the arena, corresponding to spatial decision zones. This architectural pattern suggests increased active zone-switching and turn-biased locomotion in the representative trajectory, visually indicating that caffeine combined with simulated microgravity may alter global spatial navigation and exploratory patterns in adult zebrafish, although these observations do not indicate a statistically significant synergistic effect. These visual patterns suggest differences in spatial occupancy and navigation among the representative trajectories; however, they are descriptive and should not be interpreted as evidence of statistically significant or synergistic treatment effects (Figure 4).

### 3.2. Novel Tank Test

During the novel tank test, exploratory patterns and baseline kinetic profiles were evaluated to determine how microgravitational stress and caffeine administration, both independently and in combination, influence zebrafish behavior in an anxiogenic environment.

**Figure 5:**
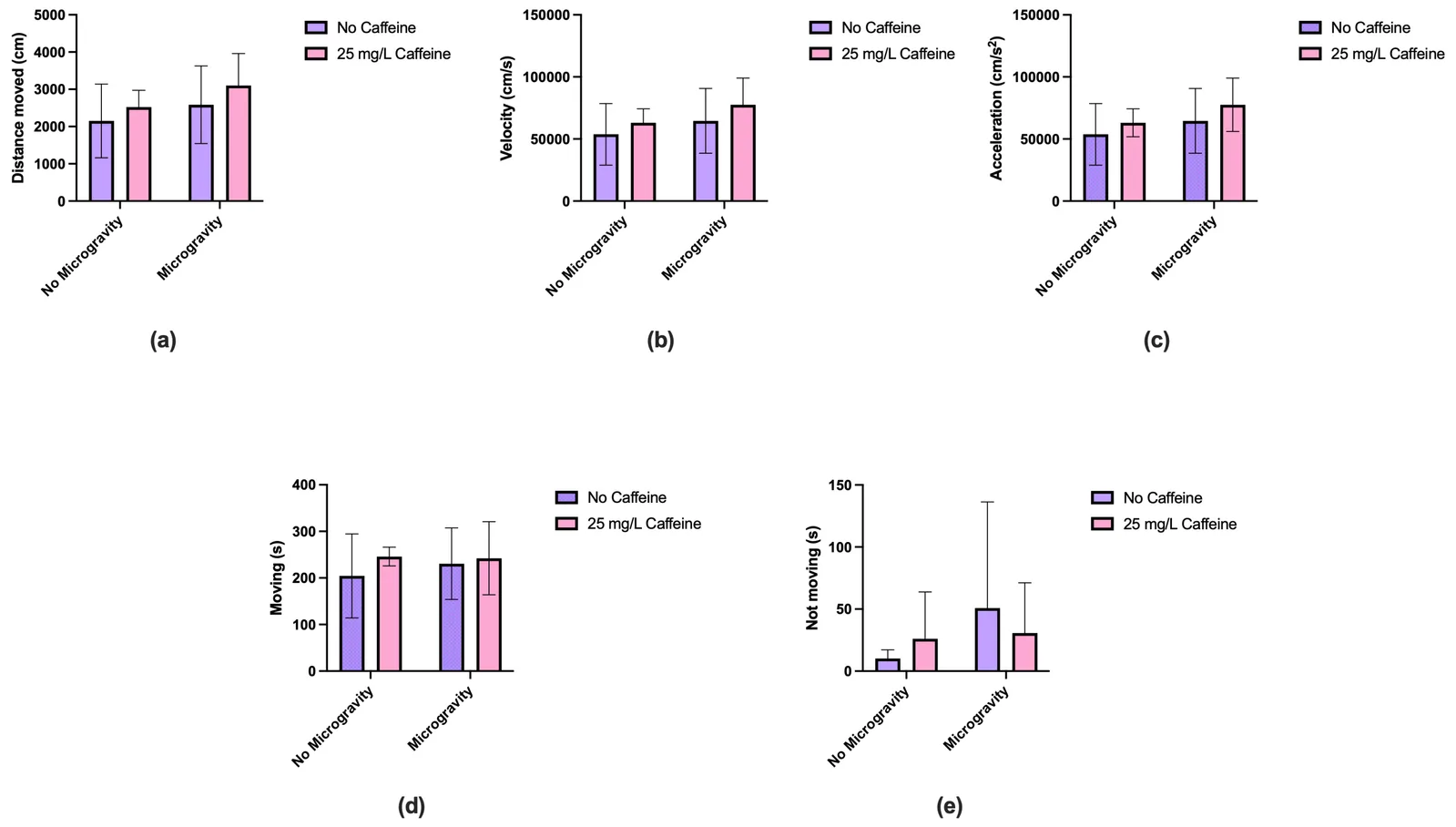
Effects of caffeine (25 mg/L) alone and in combination with microgravity on adult zebrafish locomotor and exploratory parameters during the novel tank test. Behavioral parameters measured include: (**a**) distance moved, (**b**) velocity, (**c**) acceleration, (**d**) moving duration, and (**e**) not moving duration. Data are presented as mean ± SD and were analyzed using two-way ANOVA followed by Šídák’s multiple-comparisons test.

The total distance moved was evaluated as an indicator of general locomotor activity and exploration within the novel environment (Figure 5a). Two-way ANOVA revealed no significant main effect of microgravity (*p* = 0.21) or caffeine (*p* = 0.27), and the caffeine + microgravity interaction was also not significant (*p* = 0.85). Consistently, Šídák’s multiple-comparisons test showed no significant effect of caffeine either in the absence of microgravity (*p* = 0.76) or under microgravity conditions (*p* = 0.59). Although the mean distance moved was numerically higher under microgravity and caffeine exposure, these differences were not statistically significant. Therefore, neither caffeine nor simulated microgravity, individually or in combination, significantly affected the total distance traveled during the novel tank test.

Average velocity was evaluated as an indicator of swimming intensity and overall locomotor performance during the novel tank test (Figure 5b). Two-way ANOVA revealed no significant main effect of microgravity (*p* = 0.21) or caffeine (*p* = 0.27), and the caffeine + microgravity interaction was also not significant (*p* = 0.85). Consistently, Šídák’s multiple-comparisons test showed no significant effect of caffeine either in the absence of microgravity (*p* = 0.76) or under microgravity conditions (*p* = 0.59). Although mean velocity was numerically higher in the caffeine-treated and microgravity-exposed groups, these differences did not reach statistical significance. Thus, neither caffeine nor simulated microgravity, individually or in combination, significantly altered swimming velocity during the novel tank test.

Acceleration was analyzed as an indicator of rapid changes in swimming movement and locomotor reactivity during the novel tank test (Figure 5c). Two-way ANOVA revealed no significant main effect of microgravity (*p* = 0.21) or caffeine (*p* = 0.27), and the caffeine + microgravity interaction was also not significant (*p* = 0.85). Šídák’s multiple-comparisons test further showed no significant effect of caffeine either in the absence of microgravity (*p* = 0.76) or under microgravity conditions (*p* = 0.59). Although the mean acceleration values appeared numerically higher in the microgravity and caffeine-treated groups, these differences did not reach statistical significance. Therefore, neither caffeine nor simulated microgravity, individually or in combination, significantly affected acceleration during the novel tank test.

The moving duration was quantified as a measure of active exploratory engagement during the novel tank test (Figure 5d). Two-way ANOVA revealed no significant main effect of microgravity (*p* = 0.73) or caffeine (*p* = 0.42), and the caffeine + microgravity interaction was also not significant (*p* = 0.65). Šídák’s multiple-comparisons test showed no significant effect of caffeine either in the absence of microgravity (*p* = 0.61) or under microgravity conditions (*p* = 0.96). Overall, these results indicate that neither caffeine nor simulated microgravity, individually or in combination, significantly altered the time spent in active swimming during the novel tank test.

The not moving duration was evaluated as a measure of inactivity during the novel tank test (Figure 5e). Two-way ANOVA revealed no significant main effect of microgravity (*p* = 0.34) or caffeine (*p* = 0.93), and the caffeine + microgravity interaction was not significant (*p* = 0.44). Consistently, Šídák’s multiple-comparisons test showed no significant effect of caffeine either in the absence of microgravity (*p* = 0.86) or under microgravity conditions (*p* = 0.79). These findings indicate that neither caffeine nor simulated microgravity, individually or in combination, significantly affected the duration of inactivity during the novel tank test.

**Figure 6:**
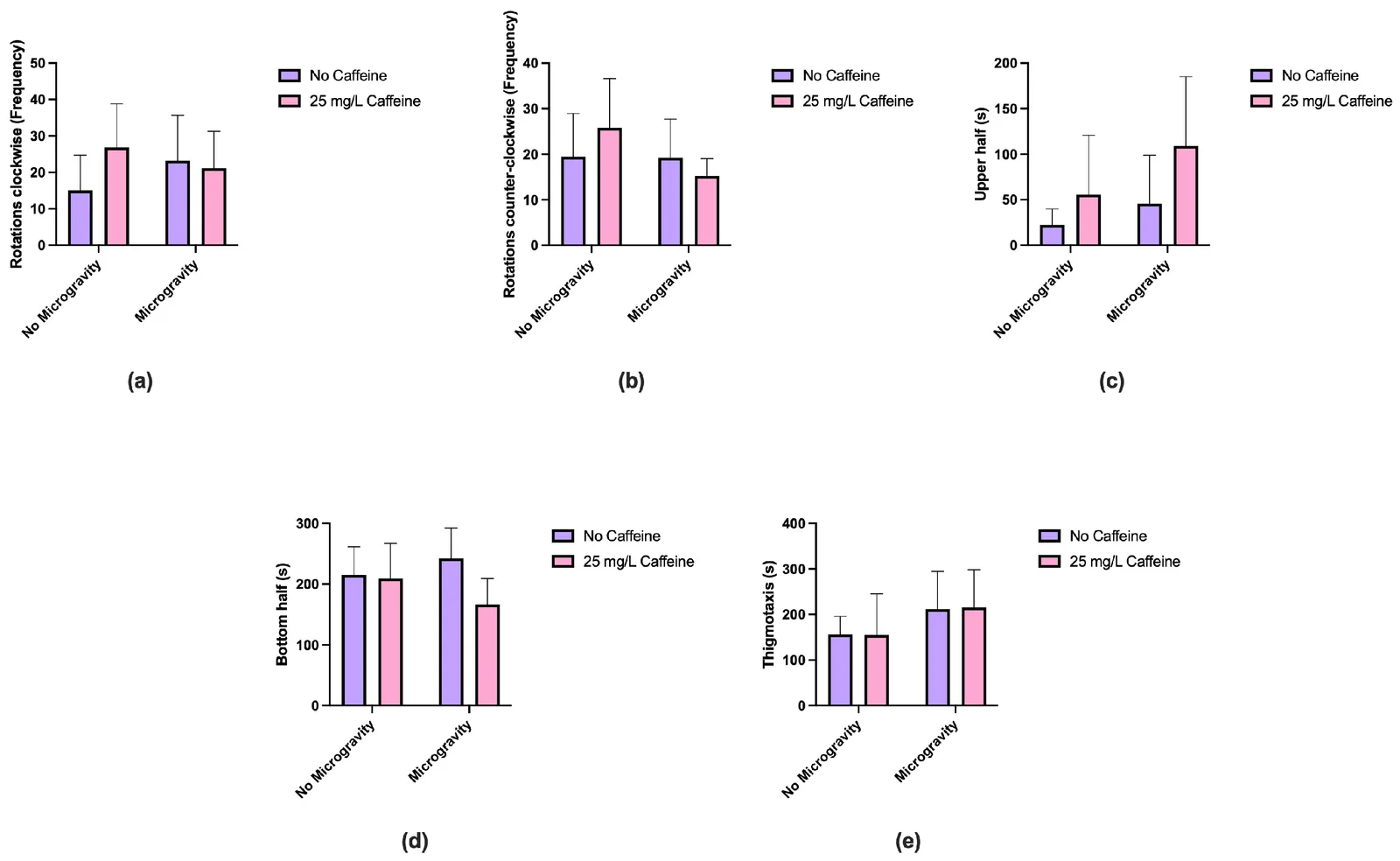
Effects of caffeine (25 mg/L) alone and in combination with microgravity on spatial navigation and zone preference parameters of adult zebrafish during the novel tank test. Behavioral parameters measured include: (**a**) clockwise rotations, (**b**) counter-clockwise rotations, (**c**) time spent in the upper half, (**d**) time spent in the bottom half, and (**e**) thigmotaxis. Data are presented as mean ± SD and were analyzed using two-way ANOVA followed by Šídák’s multiple-comparisons test.

The frequency of clockwise rotations was evaluated as a measure of directional turning behavior during the novel tank test. Two-way ANOVA revealed no significant main effect of microgravity (*p* = 0.80) or caffeine (*p* = 0.34), and the caffeine + microgravity interaction was not significant (*p* = 0.19). Consistently, Šídák’s multiple-comparisons test showed no significant effect of caffeine either in the absence of microgravity (*p* = 0.21) or under microgravity conditions (*p* = 0.95). These findings indicate that neither caffeine nor simulated microgravity, individually or in combination, significantly affected clockwise rotational behavior during the novel tank test (Figure 6a).

The frequency of counter-clockwise rotations was assessed to characterize directional turning behavior during the novel tank test. Two-way ANOVA revealed no significant main effect of microgravity (*p* = 0.18) or caffeine (*p* = 0.76), and no significant caffeine + microgravity interaction (*p* = 0.19). Consistently, Šídák’s multiple-comparisons test showed that caffeine did not significantly modify counter-clockwise rotations either under normal-gravity conditions (*p* = 0.45) or following microgravity exposure (*p* = 0.72). Although the group means showed some variation in rotational frequency, these differences were not statistically significant. Therefore, the results provide no evidence that caffeine, microgravity, or their combination significantly altered counter-clockwise rotational behavior in the novel tank test (Figure 6b).

The time spent in the upper half of the novel tank was evaluated as an indicator of vertical exploratory behavior. Two-way ANOVA revealed no significant main effect of microgravity (*p* = 0.15) or caffeine (*p* = 0.08) and no significant caffeine + microgravity interaction (*p* = 0.56). Although the caffeine main effect showed a non-significant tendency toward increased upper-half exploration, this did not reach the predefined threshold for statistical significance. Consistently, Šídák’s multiple-comparisons test showed no significant effect of caffeine under either no-microgravity conditions (*p* = 0.61) or microgravity conditions (*p* = 0.19). Thus, neither caffeine nor microgravity significantly altered upper-half exploration, and there was no statistical evidence of a synergistic effect between the two factors on this parameter (Figure 6c).

The time spent in the bottom half of the novel tank was analyzed as an indicator of bottom-dwelling behavior. Two-way ANOVA revealed no significant main effect of microgravity (*p* = 0.72) or caffeine (*p* = 0.08) and no significant caffeine + microgravity interaction (*p* = 0.13). Šídák’s multiple-comparisons test showed no effect of caffeine under no-microgravity conditions (adjusted *p* = 0.98). Under microgravity conditions, a pronounced descriptive difference was observed between the no-caffeine and 25 mg/L caffeine groups (mean values: 242.1 vs. 166.2, respectively; mean difference = 75.94); however, this comparison did not meet the predefined criterion for statistical significance (adjusted *p* = 0.05; 95% CI: −1.236 to 153.1). Therefore, although caffeine was associated with a tendency toward reduced bottom-half occupancy under microgravity, the absence of a significant caffeine + microgravity interaction provides no statistical evidence for a synergistic effect on this behavioral parameter (Figure 6d).

Thigmotaxis was analyzed as a measure of wall-associated spatial behavior. Two-way ANOVA revealed no significant main effect of caffeine (*p* = 0.97) or microgravity (*p* = 0.11) and no significant caffeine + microgravity interaction (*p* = 0.95). Although thigmotaxis was descriptively higher under microgravity conditions, microgravity accounted for 15.11% of the total variation without reaching statistical significance. Šídák’s multiple-comparisons test confirmed that caffeine did not alter thigmotaxis under either no-microgravity conditions (156.4 vs. 155.2; mean difference = 1.224; adjusted *p* > 0.99) or microgravity conditions (211.9 vs. 215.3; mean difference = −3.424; adjusted *p* > 0.99). These findings indicate that 25 mg/L caffeine did not measurably modify thigmotactic behavior and did not interact with microgravity for this behavioral endpoint (Figure 6e).

**Figure 7:**
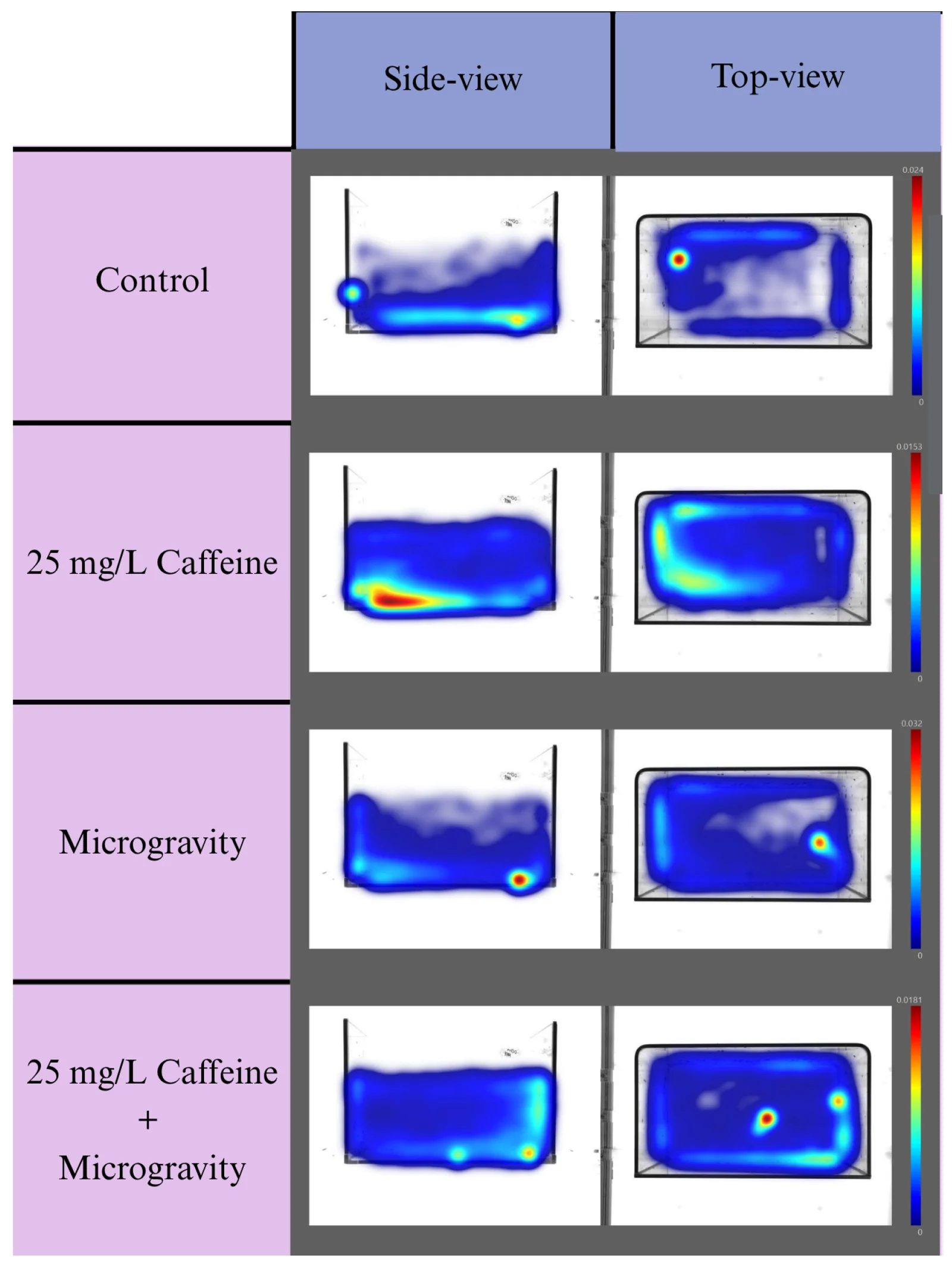
Representative dual-view EthoVision XT heat maps illustrating the spatial occupancy, geotaxis, and thigmotactic patterns of adult zebrafish during the novel tank test under the influence of 25 mg/L caffeine, microgravity, and their combination. For each group, the left panel depicts the vertical swimming profile (side-view) and the right panel depicts the horizontal exploratory layout (top-view). The color scale transitions from blue (low occupancy) to red/yellow (high occupancy).

To complement t To complement the quantitative spatial measurements, representative dual-view behavioral heat maps (side-view and top-view) were generated to evaluate the vertical distribution, geotactic responses, and wall-preference patterns of zebrafish within the novel tank arena (Figure 7). The baseline control cohort demonstrated a distinct preference for the lower region of the tank in the side-view perspective, paired with isolated, peripheral high-density spots in the top-view tracking. This spatial profile is consistent with baseline geotaxis and thigmotaxis, behavioral patterns commonly associated with anxiety-like responses in a novel environment. Acute exposure to 25 mg/L caffeine resulted in a prominent, horizontally elongated high-density hotspot (red–yellow signal) localized along the tank floor in the side-view projection, while preserving a well-defined peripheral outline in the top-view. This visual pattern suggests that caffeine was associated with horizontal locomotor activity while maintaining bottom-oriented occupancy in the representative trajectory. In contrast, the simulated microgravity group displayed a somewhat intermediate spatial distribution, with occupancy shifting toward the vertical corners of the tank, suggesting an altered exploratory pattern under simulated microgravity. Most notably, the combined 25 mg/L caffeine + microgravity cohort exhibited a visually distinct reorganization of spatial navigation. In the side-view profile, this group showed a relatively uniform and widespread vertical dispersion of tracking density, extending significantly into the upper half of the water column and suggesting greater vertical exploration in the representative trajectory. Furthermore, the top-view heat map for the co-exposure group revealed a noticeable shift in active hotspots toward the center of the arena, visually suggesting reduced wall-associated occupancy in this representative trajectory. Taken together, these visual patterns suggest differences in vertical and central arena occupancy among the representative trajectories; however, they are descriptive and should be interpreted together with the quantitative analyses, which do not provide evidence of a statistically significant caffeine-related reduction in thigmotaxis under simulated microgravity or a combined interaction between caffeine and simulated microgravity.

## 4. Discussions

Ground-based clinorotation should be interpreted as a model of simulated microgravity rather than as a direct reproduction of the microgravity environment encountered during spaceflight. Nevertheless, the use of gravity-vector-averaging systems in zebrafish is supported by previous experimental studies. In particular, Aceto et al. [20] employed a 2D clinorotation utilizing zebrafish larvae starting at 5 days post-fertilization (dpf). The authors observed that ultra-rapid transcriptomic remodeling occurs within the first 24 h of exposure. Their gene expression analyses revealed significant modifications in molecular pathways governing the musculoskeletal, cardiovascular, and nuclear receptor systems. Furthermore, prolonged exposure extending to 5 days resulted in a marked decrease in bone mineralization and overall skeletal ossification. Notably, the authors reported that systemic cortisol levels remained statistically unaltered during the rotation, demonstrating that the observed degradation of bone physiology was mediated directly by localized mechanical unloading rather than a generalized, systemic neuroendocrine stress response [20].

Carvajal-Agudelo et al. 2024 [25] evaluated the immediate and developmental impacts of 3D simulated microgravity on larval zebrafish using a Random Positioning Machine (RPM) starting at 3.5 dpf. They observed an acute, immediate reduction in cranial bone ossification across multiple developing skeletal elements upon completion of the exposure. Molecular analysis revealed that this bone loss was driven by a massive upregulation of the osteoclast-activating gene tnfsf11 (RANKL), while genes regulating bone-forming osteoblasts remained statistically unchanged, proving that the phenotype was caused by accelerated bone resorption. Crucially, the authors also observed that this structural deficit was temporary; following a 7-day recovery period in static 1 g gravity conditions, both skeletal ossification levels and resorption gene expression profiles fully returned to normal baseline values.

In a direct methodological comparison, Eaton, Carvajal-Agudelo, and Franz-Odendaal evaluated how 2D clinorotation versus 3D rotation via a Random Positioning Machine (RPM) differently impacts skeletal development in larval zebrafish. The authors observed that while both operational platforms impede normal bone development, 3D mechanical unloading causes significantly more severe and widespread inhibition of ossification than 2D rotation. Mechanistically, they discovered that 3D microgravity imposes a dual-action cellular blockade: it actively downregulates major cellular signaling pathways responsible for bone construction—specifically the WNT, BMP, and Hippo pathways—while concurrently accelerating bone-destruction signaling through the RANKL/RANK/OPG pathway [26].

Utilizing the adult zebrafish scale model—which functions as an active, miniature bone ecosystem containing living osteoblasts and osteoclasts, Carnovali et al. isolated the cellular effects of weightlessness from the physical forces generated by the simulator machinery. These studies revealed a critical confounding variable: the continuous mechanical vibration produced by the clinostat or RPM electric motors often exerts a faster, more aggressive influence on bone metabolism than the actual randomization of the gravity vector. The researchers observed that these micro-vibrations function as a powerful localized physical stressor, accelerating osteoclast enzyme activity and altering cellular signaling independent of gravitational unloading [27].

The present pilot study investigated the modulatory influence of acute caffeine administration on neurobehavioral adaptations induced by simulated microgravity in adult zebrafish. Utilizing clinostat-mediated continuous rotation to randomize the gravitational vector, we established an operational model for gravitational unloading. This setup allowed us to examine how a widely utilized dietary bioactive psychoactive compound shapes kinetic performance, stereotypic motor responses, and environmental habituation. The 25 mg/L caffeine concentration was selected based on previous behavioral studies in zebrafish; however, the present study did not include specific toxicity endpoints, and therefore this concentration should not be classified as “sub-toxic” based on the current data. The observed behavioral changes are therefore interpreted as neurobehavioral responses to caffeine and simulated microgravity rather than as evidence of beneficial or adverse effects. Our quantitative and visual findings indicate that while individual exposures to caffeine (25 mg/L) or a brief duration of simulated microgravity (1 h) induced subtle, mostly non-significant shifts in baseline kinetics, their combined application was associated with increased locomotor activity and altered spatial navigation. However, the absence of a significant interaction does not support interpretation of these combined effects as synergistic.

In the standard locomotor performance test, the combined caffeine and simulated microgravity condition was associated with increased locomotor activity, reflected by greater total distance moved (*p* = 0.04), average velocity (*p* = 0.04), and moving duration (*p* < 0.01) compared with the control condition. However, the caffeine + simulated microgravity interaction was not statistically significant for distance moved and average velocity (*p* = 0.24), indicating that these findings should not be interpreted as evidence of a synergistic effect. Moreover, increased locomotor activity cannot be considered inherently beneficial or equated with enhanced exploration, as it may also reflect psychostimulant effects, stress-related responses, or other neurobehavioral alterations. Mechanistically, caffeine acts primarily as an antagonist of central adenosine A1 and A2A receptors, thereby reducing the inhibitory actions of endogenous adenosine and modulating neurotransmitter systems involved in locomotor activity. Concurrently, simulated microgravity may alter vestibular and sensorimotor processing and act as an environmental stressor. However, because physiological stress markers and neurochemical endpoints were not assessed in the present study, the mechanisms and functional significance of the observed behavioral alterations cannot be established from the behavioral data alone.

The occurrence of increased locomotor activity in the combined exposure group suggests that simulated microgravity may influence the behavioral response to caffeine, although the absence of a significant interaction does not support a synergistic effect. This pattern was also qualitatively reflected in the multi-arm labyrinth heat maps, where co-exposed fish showed high-density residency spots at the bifurcation intersections, suggesting altered turning dynamics and spatial exploration in the representative trajectory. However, these visual patterns are descriptive and should not be considered evidence of statistically significant or synergistic treatment effects. Interestingly, acceleration rates remained statistically uniform across all cohorts (*p* > 0.05), indicating that the observed hyperlocomotion is a function of sustained exploratory velocity and reduced static pauses rather than a manifestation of sudden, uncontrolled fast kinetic bursts or startle-like escape reflexes.

Directional steering and stereotypic maneuvering also revealed asymmetric neuromodulatory effects. Acute caffeine treatment alone selectively amplified counter-clockwise rotation frequencies (*p* = 0.02), implying a potential lateralized hemispheric dopamine activation or asymmetric striatal receptor stimulation caused by standalone chemical exposure. However, when caffeine was coupled with simulated microgravity, this unilateral bias shifted toward a statistically significant surge in clockwise rotations (*p* = 0.02). Such distinct reversals in turning preference highlight how microgravity-induced vestibular disorientation alters sensorimotor coordination and integrates abnormally with caffeine’s psychostimulant properties, resulting in redesigned stereotypic search paths.

In the Novel Tank Test, neither caffeine nor simulated microgravity, individually or in combination, produced statistically significant changes in the behavioral parameters examined. Although numerical differences were observed among groups for some locomotor and spatial parameters, including immobility, vertical occupancy, and thigmotaxis, these differences did not reach statistical significance and should therefore not be interpreted as evidence of altered anxiety-related or exploratory behavior. Representative EthoVision heat maps showed descriptive differences in spatial occupancy and locomotor distribution among the experimental groups; however, these visual patterns were not subjected to independent statistical analysis and should be considered qualitative observations only. Overall, the Novel Tank Test findings indicate that the effects observed in the locomotor performance assessment were not consistently reproduced in the novel tank environment, highlighting the endpoint- and test-dependent nature of the behavioral responses observed in this pilot study.

Remarkably, the co-administration of 25 mg/L caffeine under simulated microgravity conditions appeared to reverse these stress-induced avoidance profiles. In the vertical plane, the combination group displayed a prominent visual trend toward increased exploration of the upper zone, alongside a corresponding reduction in bottom-dwelling duration (geotaxis). This vertical ascent is further illustrated by the dual-view heat maps, which revealed a uniform vertical dispersion of tracking density up through the water column and a central migration of active hotspots away from the arena walls in the top-view projection.

In zebrafish behavioral pharmacology, a transition from the bottom to the upper half of a novel arena coupled with central zone exploration signifies a reduction in anxiety-like states and an accelerated rate of environmental habituation. This implies that while a moderate dose of caffeine can sometimes act as a mild anxiogenic under normal gravity, its interaction with an active environmental stressor (microgravity) effectively switches its functional profile, shifting the behavioral output from passive, stress-induced freezing and wall-hugging to active, bold vertical exploration.

An important limitation of the present pilot study is the use of a single acute 1 h clinostat exposure. Although this exposure was sufficient to produce significant alterations in selected locomotor endpoints, the magnitude and nature of simulated-microgravity-induced behavioral responses may depend on exposure duration. Future studies should therefore employ time-course designs comparing acute, prolonged, or repeated clinostat exposures to establish the temporal profile of behavioral adaptation before evaluating pharmacological interventions.

Another limitation is that the present study focused exclusively on behavioral endpoints and did not include dedicated toxicity, physiological stress, or neurochemical assessments. Consequently, the observed increases in locomotor and rotational activity cannot be classified as beneficial or adverse based on the present data alone. Future studies incorporating toxicity and stress-related biomarkers will be necessary to determine the biological significance of these behavioral alterations.

Therefore, another limitation is the absence of a rotation-matched control. Although the experimental container was completely filled and sealed without an air–water interface, rotation may still generate hydrodynamic, mechanical, vestibular, or other sensory stimuli. Moreover, because adult zebrafish are freely swimming and capable of active body reorientation, the gravitational-vector averaging assumed at the level of the clinostat cannot be assumed to occur equivalently at the level of each individual fish. Consequently, the present design cannot determine whether the observed behavioral changes resulted specifically from gravitational-vector reorientation or, wholly or in part, from rotation-associated stimuli. The results should therefore be interpreted as responses to the clinorotation paradigm used here, rather than as effects equivalent to those of true microgravity. Future studies should incorporate an appropriate rotation-matched control and, where possible, direct characterization of hydrodynamic conditions within the exposure chamber.

Lastly, an additional limitation is the absence of a vibration-matched sham control. Because clinorotation may introduce vibration and other mechanical stimuli, the behavioral alterations observed in the present study cannot be attributed exclusively to gravity-vector averaging. Future experiments incorporating an appropriate vibration-matched control will be necessary to distinguish simulated-microgravity-associated responses from apparatus-induced mechanical effects.

As a pilot investigation, these findings indicate that caffeine and simulated microgravity are associated with distinct and endpoint-dependent behavioral responses in adult zebrafish. However, the functional significance of these behavioral alterations remains to be established. These findings provide a basis for future studies investigating the effects of caffeine and other dietary psychoactive compounds under altered-gravity conditions, using larger sample sizes, additional physiological and neurochemical endpoints, and longer exposure periods.

## 5. Conclusions

In conclusion, this pilot study indicates that acute exposure to simulated microgravity using a clinostat produces selective behavioral alterations in adult zebrafish and that caffeine modifies behavioral responses under these experimental conditions. Simulated microgravity alone was associated with significant effects on specific locomotor performance endpoints, including total distance moved, average swimming velocity, and clockwise rotational behavior, whereas these effects were not consistently observed across all behavioral assessments.

In the locomotor performance test, the combined caffeine and simulated microgravity condition was associated with increased locomotor activity, reflected by higher distance moved, swimming velocity, and moving duration compared with the control condition. However, because the caffeine + simulated microgravity interaction was not statistically significant for these endpoints, these findings should be interpreted as combined treatment effects rather than definitive evidence of a synergistic interaction.

In contrast, during the Novel Tank Test, simulated microgravity and caffeine did not produce statistically significant differences in the principal quantitative behavioral parameters. Nevertheless, representative EthoVision heat maps qualitatively suggested alterations in spatial occupancy and locomotor distribution following clinostat exposure and the combined treatment, supporting the presence of descriptive changes in exploratory patterns that warrant further investigation.

Overall, these findings support the usefulness of adult zebrafish as a model for investigating behavioral responses to ground-based simulated microgravity and pharmacological exposure. Since clinostat culture represents a simulation of gravity-vector averaging rather than true space microgravity, and considering the pilot nature of this study, further experiments with larger sample sizes and complementary spaceflight or alternative simulated-microgravity models are necessary to clarify the mechanisms underlying the observed behavioral responses and to evaluate the potential role of caffeine in altered-gravity environments.

## Data Availability

The datasets generated and analyzed during the current study are available from the corresponding author upon reasonable request.
